# Multi-domain training in healthy old age: Hotel Plastisse as an iPad-based serious game to systematically compare multi-domain and single-domain training

**DOI:** 10.3389/fnagi.2015.00137

**Published:** 2015-07-23

**Authors:** Julia C. Binder, Jacqueline Zöllig, Anne Eschen, Susan Mérillat, Christina Röcke, Sarah F. Schoch, Lutz Jäncke, Mike Martin

**Affiliations:** ^1^Division of Gerontopsychology and Gerontology, Department of Psychology, University of ZurichZurich, Switzerland; ^2^International Normal Aging and Plasticity Imaging Center, University of ZurichZurich, Switzerland; ^3^University Research Priority Program “Dynamics of Healthy Aging”, University of ZurichZurich, Switzerland; ^4^Division of Neuropsychology, Department of Psychology, University of ZurichZurich, Switzerland

**Keywords:** healthy aging, cognitive decline, cognitive training, multi-domain training, serious game, iPad

## Abstract

Finding effective training interventions for declining cognitive abilities in healthy aging is of great relevance, especially in view of the demographic development. Since it is assumed that transfer from the trained to untrained domains is more likely to occur when training conditions and transfer measures share a common underlying process, multi-domain training of several cognitive functions should increase the likelihood of such an overlap. In the first part, we give an overview of the literature showing that cognitive training using complex tasks, such as video games, leisure activities, or practicing a series of cognitive tasks, has shown promising results regarding transfer to a number of cognitive functions. These studies, however, do not allow direct inference about the underlying functions targeted by these training regimes. Custom-designed serious games allow to design training regimes according to specific cognitive functions and a target population's need. In the second part, we introduce the serious game Hotel Plastisse as an iPad-based training tool for older adults that allows the comparison of the simultaneous training of spatial navigation, visuomotor function, and inhibition to the training of each of these functions separately. Hotel Plastisse not only defines the cognitive functions of the multi-domain training clearly, but also implements training in an interesting learning environment including adaptive difficulty and feedback. We propose this novel training tool with the goal of furthering our understanding of how training regimes should be designed in order to affect cognitive functioning of older adults most broadly.

## Multi-domain training in healthy old age

Normal aging occurs along with declines in executive functions, processing speed, reasoning, and episodic memory roughly from 65 years of age (for reviews see e.g., Salthouse, 2010; Schaie, 2012). A large proportion of our society now grows older than 65 years and lives longer in the last phase of life due to increasing life expectancy (Cauley, 2012). Thus, there is great interest in and need for interventions that counteract age-related cognitive decline and possibly extend the time during which everyday life can be mastered independently. Different training approaches have been successful in improving the trained functions, but generalization to different contexts or cognitive functions has been limited (for a review see e.g., Lustig et al., 2009). This pattern of findings has led to vivid discussions about how training programs should be designed to enable the training to transfer to other cognitive functions or daily life (e.g., Eschen, 2012; Green et al., 2014; Karbach and Verhaeghen, 2014; Noack et al., 2014). In recent years, multi-domain training has emerged as a promising training approach that uses interesting and complex learning environments (Park et al., 2007; Green and Bavelier, 2008; Karbach, 2014; Stine-Morrow et al., 2014). Multi-domain training combines several cognitive functions and demands their interplay, thereby simulating real-life demands more closely than single-domain training (Green and Bavelier, 2008; Lustig et al., 2009).

Training transfer is defined as the extent to which the trained functions improve performance on tasks targeting similar or different cognitive functions. Transfer is considered small, medium, or large depending on the similarity or distance of the training to the transfer tasks (for a discussion see Noack et al., 2014). It is proposed that transfer is mediated by the extent to which both training and transfer tasks depend on the same cognitive processes, brain structures, or both (Jonides, 2004; Dahlin et al., 2008; Lustig et al., 2009; Kuwajima and Sawaguchi, 2010; Buschkuehl et al., 2012; Taatgen, 2013). Based on these considerations, training higher order cognitive functions, such as executive functions or working memory, is highly promising because these functions underlie a wide range of performance-relevant cognitive domains (Karbach and Verhaeghen, 2014). Likewise, training a range of different cognitive functions has the potential for broader transfer since this increases the probability that one of the training domains overlaps with another cognitive function or task. Furthermore, the simultaneous training of several cognitive functions has the potential to train not only each single function, but also cognitive functions that coordinate their simultaneous administration. Although such multi-domain training has shown promising initial results (Hertzog et al., 2008), there is only a limited number of such studies to date. We first give an overview of multi-domain training studies with a focus on healthy older adults. A systematic evaluation of the existing multi-domain training studies is complicated by the fact that training protocols vary greatly. We therefore broadly divide multi-domain training into three groups. (1) One group of multi-domain training studies that introduce participants to novel leisure activities, (2) a second group of studies that train several cognitive functions and health-related domains sequentially, and (3) a third group of studies that consist of video or computer game training. Second, we present a novel iPad-based training tool specifically developed to systematically compare multi-domain and single-domain training.

### Multi-domain training with complex leisure activities

Several interventions experimentally introduced older adults to new, mentally stimulating, and complex leisure activities. They are based on the findings that an active lifestyle in old age is generally associated with reduced age-related cognitive decline (e.g., Hultsch et al., 1999). Experimental interventions consistently showed improvements of healthy older adults' cognition when leisure activity groups were compared to passive control groups or control activities that were not mentally stimulating (for reviews see Park et al., 2007; Hertzog et al., 2008). In general, these studies have shown good acceptance in older adults (see e.g., Parisi et al., 2007; Fried et al., 2013).

#### Comparison of multi-domain leisure activity training to no training

Two community-based interventions assigned older adults to stimulating environments, which this demographic typically does not engage in anymore. In the Experience Corps program (Fried et al., 1997, 2004, 2013; Carlson et al., 2008, 2009), older adults volunteered in public elementary schools to support students from kindergarten through third grade. Participants were randomly assigned to either a wait-list control or an intervention group. The intervention group underwent an intense 2-week training and instruction phase and was then placed into a school where they volunteered in different roles (e.g., supporting literacy development, helping children find library books, fostering conflict resolution skills) for at least 15 h over 3–4 days a week during a 9-month school year. Participants in the intervention group reported increased physical, social, and cognitive activity levels (Fried et al., 2004) and showed improvements in memory and executive function (Carlson et al., 2008), while there was a slight decrease in the wait-list control group (for study details see Table 1). In the Senior Odyssey program (Stine-Morrow et al., 2008), participants prepared a tournament that consisted of on-site challenges that had to be solved spontaneously (problem solving tasks or handicrafts) and long-term problems that had to be prepared in a 6-month preparation phase of 20 group meetings led by a coach (e.g., presenting a new interpretation of a classical piece of literature). Participants were randomly assigned to preparing for the tournament or to a passive control group. In general, intervention-related effects were small: participants in the intervention group showed better performance on processing speed, reasoning, and fluency, and on the composite score of fluid ability (Gf), while there were no improvements on working memory and visuo-spatial processing (see Table 1). Fluid ability improvements have also been reported in a third study that compared an intervention group completing creative tasks at home (e.g., creative drawing, modeling, word-logic puzzles, identification of mystery photos) to a control group that attended a few social meetings over 10–12 weeks (see Table 1; Tranter and Koutstaal, 2008). These exemplar programs show that healthy older adults participating in complex leisure interventions can improve their fluid abilities, executive functions, or memory when compared to passive or active control groups (Carlson et al., 2008; Stine-Morrow et al., 2008; Tranter and Koutstaal, 2008). However, the crucial question is which activities benefit cognition most.

**Table 1:** **Summary of training studies with complex leisure activities**.

**Study**	**Intervention/training groups (TG) and control groups (CG)**	**Age (years)**	***N***	**Training duration**	**Measurement time points and outcome measures**	**Training-related effects**
Fried et al., 2004; Carlson et al., 2008	Experience Corps group (volunteer work in schools, team meetings of volunteers; TG), Passive control group (pCG)	60–86 *M* = 69	Total *n* = 128 (post *n* = 110) TG: *n* = 70 (post *n* = 62) pCG: *n* = 58 (post *n* = 48)	4–8 months: Introduction of 2 weeks (30 h/week), volunteer work of 15 h/week Total training: >500 h	Pre, post -Self-reports: physical, social, and cognitive activity - Executive function - Memory - Psychomotor speed	*t*-Tests/ANCOVAs: TG improved physical, social, and cognitive ability levels assessed with self- and interviewer-administered questionnaires, executive function, and memory (no effect sizes provided).
Noice et al., 2004	Acting (TG1), Visual arts (TG2), Passive control group (pCG)	60–86 *M* = 74	Total *n* = 111 TG1: *n* = 44 TG2: *n* = 36 pCG: *n* = 31	4 weeks: 2 sessions/week for 1.5 h (total 7 sessions) Total training: ca. 10.5 h	Pre, post 4-week follow-up4-month follow-up (only TG1) - Free recall (immediate, delayed) - Working memory - Problem solving - Self-esteem - Psychological well-being	MANCOVAs/ANOVAs: Significant group effects at post-test for recall (η^2^ = 0.07), problem solving (η^2^ = 0.25), and psychological well-being (η^2^ = 0.13), contrasts revealed that the TG1 performed better as pCG on recall, problem solving, working memory (marginally significant) and showed higher psychological well-being; compared to TG2, TG1 showed better performance on problem solving and higher psychological well-being. TG1 maintained training-related changes.
Noice and Noice, 2009	Acting (TG1), Singing (TG2), Passive control group (pCG)	68–93 *M* = 82	Total *n* = 122 TG1: *n* = 42 TG2: *n* = 40 pCG: *n* = 40	4 weeks: 2 sessions/week for 1 h (total 8 sessions) + homework Total training: 8 h + homework	Pre, post - Free recall (immediate, delayed) - Working memory - Fluency - Problem solving - Personal growth - Memory controllability - Life style activities	MANCOVAs/ANCOVAs: Significant group effects at post-test for immediate ($\eta_{\text{p}}^{2}=0.21$) and delayed recall ($\eta_{\text{p}}^{2}=0.07$), fluency ($\eta_{\text{p}}^{2}=0.18$), and problem solving ($\eta_{\text{p}}^{2}=0.27$). TG1 performed better on all of these measures compared to TG2 and pCG.
Park et al., 2014	Productive engagement - Digital photography (TG1) - Quilting (TG2) - Dual (TG3) Receptive engagement - Social group (aCG1) - Placebo group (aCG2)	60–90 *M* = 72	Total *n* = 221 TG1: *n* = 29 TG2: *n* = 35 TG3: *n* = 42 aCG1: *n* = 36 aCG2: *n* = 39	14 weeks: 15 h/week (5 h of structured activities, 10 h self-directed) Total training: ca. 210 h	Pre, post - Processing speed - Mental control - Episodic memory - Visuo-spatial abilities	ANOVAs (group × time interactions): TG1 vs. aCG2: TG1 improved more in episodic memory (*d* = 0.54) and in visuo-spatial processing (*d* = 0.29). TG3 vs. aCG2: TG3 improved more in episodic memory (*d* = 0.22) and processing speed (*d* = 0.29).
Stine-Morrow et al., 2008	Senior Odyssey (TG), Passive control group (pCG)	58–93 *M* = 73	Total *n* = 150 TG: *n* = 87 (post *n* = 64) pCG: *n* = 63	6 months: 20 weekly meetings ( *M* = 16 group meetings, range 6–20) Total training: >20 h	Pre, post - Processing speed - Working memory - Reasoning - Visuo-spatial processing - Fluency - Gf (composite) - Mindfulness - Need for cognition - Memory self-efficacy	*t*-Tests (group differences in change): TG shows small effects for processing speed, reasoning, fluency, and on the overall fluid ability composite (Gf) score (exact effect sizes are not reported, change is calculated separately per group). TG showed no differential effects on mindfulness, need for cognition, memory self-efficacy.
Tranter and Koutstaal, 2008	Different home + group activities (TG), Social group (aCG)	60–75 *M* = 68	Total *n* = 44 TG: *n* = 22 aCG: *n* = 22	10–12 weeks: 2 sessions/week for 1 h (total of 12 different home activities) + 3 group meetings, number of social group meetings for aCG not specified Total training: not specified	Pre, post - Gf (Cattell's Culture Fair intelligence test) - Visuo-spatial ability	ANOVAs (group × time interactions): TG improved more on Gf (*d* = 0.56) and similarly on visuo-spatial ability (no effect size provided)

*aCG, active control group; pCG, passive control group (to simplify wait-list control groups are counted as passive control groups); Gf, fluid intelligence. Effect sizes were taken from original articles and classified according to the following conventions (Lakens, 2013): Eta squared and partial eta squared (η^2^/$\eta_{p}^{2}$): small effect: η^2^/$\eta_{p}^{2}$ = 0.01; medium effect: η^2^/$\eta_{p}^{2}$ = 0.06; large effect: η^2^/$\eta_{p}^{2}$ = 0.14. Cohen's d (Cohen, 1992): small effect: d = 0.20; medium effect: d = 0.50; large effect: d = 0.80*.

#### Comparison of different types of multi-domain leisure activity training

Two studies have ventured to answer this question and compared different types of leisure activities to each other. For example, in the Synapse Project, older adults learned complex new skills such as digital photography, quilting, or both (Park et al., 2014). These three groups were compared to a group that took part in social activities (social group) and a group that engaged in placebo activities at home that were not supposed to specifically enhance cognition (e.g., watching television, listening to music etc.). Pre- and post-intervention, participants completed a test battery assessing processing speed, mental control, episodic memory, and visuo-spatial processing. Comparing the three intervention groups of complex new skills to the two control conditions (social and placebo group), participants in the first intervention types showed significantly higher improvements on episodic memory. When comparing each intervention group to the placebo group separately, the highest improvements were found in the digital photography group, with a medium effect on episodic memory and a small effect on visuo-spatial processing. The dual condition, which consisted of engaging in digital photography and quilting half of the time each, showed small effects on episodic memory and processing speed. The quilting group did not show improvements on any of the cognitive measures (Park et al., 2014; see Table 1). In the second comparative study, Noice et al. (2004) randomized participants to an acting or a visual arts class. They met their assigned group for a total of eight sessions twice a week over 4 weeks. Consistent with the assumption that acting is more demanding, participants in the acting class outperformed the arts group and a passive control group on problem solving and psychological well-being (medium to large group effects; see Table 1). The acting group additionally outperformed the passive control group on memory recall. Training-related improvements were stable throughout the 4-month follow-up. These results were replicated in a follow-up study comparing an acting class to a singing class (Noice and Noice, 2009).

Overall, the outlined studies varied greatly in the type of activities and in the intervention duration (4 weeks to 8 months with varying training intensity from a total of 10 h up to more than 500 h; see Table 1). Depending on the type of leisure activities, cognition improved differentially and effect sizes ranged from small to large with effects on transfer tasks typically very different from the intervention. However, it is difficult to infer which cognitive functions were involved in the training activities.

### Multi-domain training with a series of different tasks

Another approach to increase the breadth of training transfer is to train several cognitive functions sequentially. These studies use training tasks that are administered in series, training either well-defined cognitive functions (Schmiedek et al., 2010a; Cheng et al., 2012; Chambon et al., 2014) or more complex tasks similar to leisure activities (Winocur et al., 2007a).

The intervention by Stuss et al. (2007), for example, used rather complex tasks and comprised three training modules: memory strategy training, goal management training, and psychosocial training to enhance self-esteem and positive attitudes toward age-related changes. The training modules were administered in a fixed order, each during 4 weeks with a weekly 3-h interactive group session and an additional hour of homework to apply the learning content to everyday life. Participants were randomly assigned to an early and a late training group (wait-list within-subjects design). Outcome measures of the three intervention domains were assessed at pretest, posttest, and at a 6-month follow-up. There were medium to large intervention-related effects on memory (Craik et al., 2007), large improvements in organizational real-life tasks and self-reported executive problems attributed to the goal management module (Levine et al., 2007), and a medium increase of psychosocial well-being (Winocur et al., 2007b). Some training-related improvements of the three modules were maintained at the 6-month follow-up (see Table 2; Levine et al., 2007; Winocur et al., 2007b).

**Table 2:** **Summary of training studies with a series of different tasks**.

**Study**	**Intervention/training groups (TG) and control groups (CG)**	**Age (years)**	***N***	**Training duration**	**Measurement time points and outcome measures**	**Training-related effects**
Chambon et al., 2014	Attention and memory training group (TG), Leisure group (aCG), Passive control group (pCG)	*M* = 74	Total *n* = 45 TG: *n* = 15 aCG: *n* = 15 pCG: *n* = 15	12 weeks: 2 session/week for 1 h (total 24 sessions) Total training: 24 h	Pre, post, 6-month follow-up - Recognition - Free recall (immediate and delayed) - Cued recall (immediate and delayed) - Memory self-perception - Self-esteem	ANOVAs (group × time interactions): TG improved more on recognition ($\eta_{\text{p}}^{2}=0.14$–0.18), immediate ($\eta_{\text{p}}^{2}=0.14$–0.34) and delayed ($\eta_{\text{p}}^{2}=0.17$–0.24) free recall, and self-perception of memory functioning ($\eta_{\text{p}}^{2}=0.66$).
Cheng et al., 2012	Multi-domain (mTG: reasoning, memory, problem solving, visuo-spatial map reading, handcraft, physical exercise), Single domain (sTG: reasoning), Passive control group (pCG)	65–75 *M* = 70	Total *n* = 270 (post-training: *n* = 173) mTG: *n* = 90 (post: 54) sTG: *n* = 90 (post: 59) pCG: *n* = 90 (post: 60)	12 weeks: 2 sessions/week for 1 h (24 training sessions) Total training: 24 h	Pre, post, 6-/12-month follow-up - General cognitive abilities - Reasoning - Memory - Visuo-spatial abilities - Language - Attention - Processing speed	ANOVAs (group × time interactions):Posttest: mTG and sTG improved more on reasoning compared to pCG (mTG: *d* = 0.53, sTG: *d* = 0.52); mTG improved more on immediate (mTG: *d* = 0.53) and delayed (mTG: *d* = 0.51) memory than sTG; sTG improved more on visuo-spatial abilities (*d* = 0.36) than mTG. Follow-up: mTG and sTG showed stable effects for reasoning at 6-month follow-up (mTG: *d* = 0.47, sTG: *d* = 0.48), while only mTG showed a stable effect at the 12-month follow-up (*d* = 0.46); mTG showed a stable effect of small effect size for delayed memory at the 12-month follow-up (*d* = 0.39); sTG showed a stable effect for visuo-spatial abilities at the 6-month follow-up (*d* = 0.40).
Craik et al., 2007; Levine et al., 2007; Stuss et al., 2007; Winocur et al., 2007b	Within-subjects design: early training group (eTG) vs. late training group (lTG) with 3 modules: - Memory training - Goal management training - Psychosocial training	71–87 *M* = 79	Total *n* = 49 eTG: *n* = 29 lTG:: *n* = 20	12 weeks (4 weeks/module): 1 session/week for 3 h + 1 h home work/week Total training: 48 h	Pre, post, 6-month follow-up - Memory - Fluency - Goal management - Psychosocial: dysexecutive function test, everyday activities, locus of control, life orientation scale, happiness	ANCOVAs: eTG vs. lTG (= pCG) eTG improved more on immediate (η^2^ = 0.18) and delayed recall (η^2^ = 0.10), secondary memory (η^2^ = 0.08), psychosocial well-being (η^2^ = 0.12), and goal-management (η^2^ = 0.24).
Schmiedek et al., 2010b	Training of processing speed, episodic memory, and working memory (TG), Passive control group (pCG)	65–81 *M* = 71	Total *n* = 142 TG: *n* = 103 pCG: *n* = 39 (+ younger participants)	6 months: up to 6 sessions/week: 100 sessions for 1 h (total ca. 100 sessions) Total training: 100 h	Pre, post - Near and far working memory - Episodic memory - Processing speed - Reasoning	Mixed models (group × time interactions)/latent difference score models: TG showed near (animal span: *d* = 0.42) and far transfer (rotation span: *d* = 0.60) on working memory, transfer to the latent factor of near working memory (*d* = 0.31), transfer to reasoning (Raven: *d* = 0.54) and episodic memory (word pairs: *d* = 0.50).

*aCG, active control group; pCG, passive control group (to simplify wait-list control groups are counted as passive control groups). Means for age are indicated when the ranges were not reported. Effect sizes were taken from original articles and classified according to the following conventions (Lakens, 2013): Eta squared and partial eta squared (η^2^/$\eta_{p}^{2}$): small effect: η^2^/$\eta_{p}^{2}$ = 0.01; medium effect: η^2^/$\eta_{p}^{2}$ = 0.06; large effect: η^2^/$\eta_{p}^{2}$ = 0.14. Cohen's d (Cohen, 1992): small effect: d = 0.20; medium effect: d = 0.50; large effect: d = 0.80*.

In one of the most intensive training studies, the COGITO study (Schmiedek et al., 2010a,b), younger and older adults trained episodic memory, working memory, and processing speed. Each cognitive function was trained by several computerized training tasks with a fixed difficulty level. Training took place in 100 one-hour training sessions over approximately 6 months and was compared to a passive control group. Performance on untrained transfer tests that assessed reasoning in addition to the trained functions was tested at baseline and post-test. Older adults showed a small effect on a latent factor of near working memory transfer. On the single task level, training resulted in near and far transfer effects on working memory (with a small and medium effect size, respectively), a medium transfer effect on episodic memory and a medium transfer effect on reasoning. No effects were found for processing speed (see Table 2; Schmiedek et al., 2010b). These findings regarding transfer effects on episodic memory are in line with those of a recent attention and memory training study (see Table 2; Chambon et al., 2014).

There is one randomized controlled study that directly compared the sequential training of several cognitive functions to the training of only one of these functions (Cheng et al., 2012). Participants in the two intervention groups either trained only one function, namely reasoning (single-domain training), or several cognitive functions (multi-domain training of reasoning, memory, problem solving, visuo-spatial map reading, handcraft, and physical exercise) for an hour twice a week over 12 weeks. Training difficulty was increased, but could not be adjusted in a fine-grained manner to each individual's performance since paper and pencil tasks were used and training took part in group sessions. Immediately after training, both intervention groups showed training-related improvements of medium effect size on an outcome measure of reasoning when compared to the passive control group. This effect was maintained at the 6-month follow-up in both groups. Contrary to expectations, only the multi-domain training group showed maintenance at the 12-month follow-up although these participants trained reasoning considerably less intensively than the participants of the single-domain (reasoning) training group. Results for other outcome measures were mixed (see Table 2; Cheng et al., 2012).

In sum, findings from studies comparing the effects of training a series of several cognitive functions are mixed. While the three training modules used in the intervention by Winocur et al. (2007a) resulted in improvements on all three training domains, other studies could find improvements on only some of the trained functions (Schmiedek et al., 2010b; Cheng et al., 2012; Chambon et al., 2014) and therefore did not necessarily show broad cognitive improvements. However, a general conclusion about the breadth of transfer is not possible due to the heterogeneity of the studies and a systematic overview is difficult since the available studies fail to share common study features. This is also true for video and computer game training studies which are reviewed next, but their defining feature is the virtual training environment.

### Multi-domain training with video and computer games

A recent meta-analysis (Toril et al., 2014) showed that video game training independent of its type (commercial action video games, simple computer games, brain training designed to enhance cognition) has a beneficial effect on overall cognitive functioning of healthy older adults. Mean effects across all studies were small to medium for memory, attention, and reaction time. There was no evidence for an effect on executive function (see Table 3). However, considering individual studies, video game training resulted in small to moderate effects on executive function (e.g., Basak et al., 2008). Generally, age and duration of training significantly moderated the effects on cognitive functions, with older adults benefitting more (71–80 vs. 60–70 years) and shorter interventions being more effective (1–6 vs. 7–12 weeks; Toril et al., 2014). Several training studies were conducted with commercial video games whose primary purpose is entertainment (e.g., Basak et al., 2008; Stern et al., 2011). In contrast, brain-training programs and serious games have been developed and specifically designed for training cognition rather than for mere entertainment purposes (e.g., Ackerman et al., 2010; Anguera et al., 2013).

**Table 3:** **Summary of training studies with video and computer games**.

**Study**	**Intervention/training groups (TG) and control groups (CG)**	**Age (years)**	***N***	**Training duration**	**Measurement time points and outcome measures**	**Training-related effects**
Ackerman et al., 2010	Wii Brain Academy, Reading assignments (within-subjects design, counterbalanced)	50–71 *M* = 61	Total *n* = 78	8 weeks (4 weeks for each assignment: Wii and Reading): 5 sessions/week for 1 hTotal training: 40 h (20 h per condition)	Pre, mid, post - Gc - Gf - Processing speed	ANOVAs: No condition × time interaction
Anguera et al., 2013	Neuroracer dual (mTG), Neuroracer single (sTG), Passive control group (pCG)	60–85 *M* = 67	Total *n* = 46 mTG: *n* = 16 sTG: *n* = 15 pCG: *n* = 15	4 weeks: 3 sessions/week for 1 hTotal training: 12 h	Pre, post, 6-month follow-up - Working memory - Sustained attention - Dual-tasking - Useful field of view - Visual working memory capacity - Processing speed	ANOVAs (group × time interactions): Significant group × time (pre-post) interactions for working memory (mTG > sTG: *d* = 0.42–0.67; mTG > pCG: *d* = 0.78–0.98) and sustained attention (mTG > sTG: *d* = 0.46–0.54; mTG > pCG: *d* = 0.75—0.98). Some non-significant improvements for dual-tasking (mTG > sTG: *d* = 0.27; mTG > pCG: *d* = 0.35), useful field of view (mTG > sTG: *d* = 0.68; mTG > pCG: *d* = 0.02), visual working memory capacity (mTG > sTG: *d* = 0.05–0.15; mTG > pCG: *d* = 0.11–0.54), processing speed (mTG > sTG: *d* = 0.14–0.22; mTG > pCG: *d* = 0.32–0.51)
Basak et al., 2008	Strategy video game Rise of Nations (TG), Passive control group (pCG)	*M* = 69	Total *n* = 39 TG: *n* = 19 pCG: *n* = 20	4–5 weeks: 3 sessions/week for 1.5 h (total 15 sessions)Total training: 23.5 h	Pre, mid, post - Executive control - Visuo-spatial abilities	ANOVAs (group × time interactions): Task switching (reduced switch costs for TG: η^2^ = 0.10), working memory (η^2^ = 0.10), visual short-term memory (η^2^ = 0.09), reasoning (η^2^ = 0.11), mental rotation (η^2^ = 0.05)
Peretz et al., 2011	CogniFit Personal Coach® (TG1), Non-adaptive computer games (TG2: e.g., Tetris, puzzles, math, memory pairs)	*M* = 68	Total *n* = 121 TG1: *n* = 66 TG2: *n* = 55	3 months: 2–3 sessions/week for 20–30 min (total of 24 sessions) Total training: ca. 12 h	Pre, post - Attention - Memory - Working memory - Reasoning - Planning	Mixed models: Group × time interactions: TG1 improved significantly more on focused attention (TG1: *d* = 0.63; CG: *d* = 0.29), visuo-spatial working memory (TG1: *d* = 0.43), visuo-spatial learning (TG1: *d* = 0.51). Effect sizes were taken from (Kueider et al., 2012)
Toril et al., 2014	Meta-analysis	60–80	Total *n* = 913 TG: *n* = 474 CG: *n* = 439	1–12 weeks	Pre, post - Global cognition - Memory - Attention - Reaction time - Executive function	Global cognition (*d* = 0.38, CI = 0.13–0.62) Memory (*d* = 0.39, CI = 0.01–0.64) Attention (*d* = 0.37, CI = 0.17–0.57) Reaction time (*d* = 0.63, CI = 0.42–0.84) Executive function (*d* = 0.16, CI = −0.10–0.42)
Whitlock et al., 2012	World of Warcraft (TG), Passive control group (pCG)	60–77 *M* = 68	Total *n* = 39 TG: *n* = 19 CG: *n* = 20	2 weeks: 1 h/dayTotal training: 14 h	Pre, post - spatial ability - processing speed - attentional control - reasoning - memory	ANOVAs: TG improved more than pCG in attentional control (η^2^ = 0.10).

*aCG, active control group; pCG, passive control group (to simplify wait-list control groups are counted as passive control groups); Gf, fluid intelligence; Gc, crystallized intelligence. Means for age are indicated when the ranges were not reported. CI, confidence interval; Effect sizes were taken from original articles and classified according to the following conventions (Lakens, 2013): Eta squared and partial eta squared (η^2^/$\eta_{p}^{2}$): small effect: η^2^/$\eta_{p}^{2}$ = 0.01; medium effect: η^2^/$\eta_{p}^{2}$ = 0.06; large effect: η^2^/$\eta_{p}^{2}$ = 0.14. Cohen's d (Cohen, 1992): small effect: d = 0.20; medium effect: d = 0.50; large effect: d = 0.80*.

*Commercial action video games* have been the focus of one line of research interested in the effects of complex training experiences because of their high perceptual, cognitive, and motor loads that challenge different cognitive functions simultaneously. Interventions with action video games have indeed been shown to improve a wide range of cognitive functions such as attentional control, multitasking, and mental rotation (Bavelier et al., 2012; Green and Bavelier, 2012). Therefore, it has been proposed that action video games provide a learning environment that does not primarily foster game-specific learning, but rather enhances the ability to extract relevant information from new environments and adapt flexibly to them, a process termed “learning to learn” (cf. Bavelier et al., 2012). For example, the action video game Rise of Nations increased older adults' performance on task switching, working memory, reasoning, visual short-term memory, and mental rotation after 23.5 h of total gaming time compared to a passive control group (see Table 3; Basak et al., 2008).

*Serious games* refer to custom-designed games with the primary purpose of improving health or imparting new knowledge in various age groups (for reviews and taxonomy of serious games see Rego et al., 2010; Wiemeyer and Kliem, 2012; Robert et al., 2014). Serious games that specifically target age-related decline in healthy adults and persons suffering from mild cognitive impairment or Alzheimer's disease are in the early stages of development (Fua et al., 2013; Robert et al., 2014). The serious game Neuroracer (Anguera et al., 2013) was designed in such a way that a dual-task training of visuomotor function and signal detection could be compared to the training of each of its components. Healthy older adults either trained with the combined task of virtually driving a car on a road and simultaneously reacting to signs as quickly as possible or practiced both task components individually in series, each for half of the total training time. Training took place for 1 h three times a week over 4 weeks (i.e., 12 training sessions). Both training groups improved performance in the two training tasks (driving the car and reacting to signs), but only the dual-task training group improved performance on the trained simultaneous dual-task condition. Furthermore, participants of the dual-task training showed transfer to a working memory and a sustained attention task (see Table 3; Anguera et al., 2013).

#### Commercial computer games vs. custom-designed serious training games

The meta-analysis of Toril et al. (2014) did not find an overall difference between commercial computer games and custom-designed serious training games. Nevertheless, a comparison of training with several classic computer games to training with several adaptive cognitive tasks from the brain-training program CogniFit Personal Coach® revealed that the brain-training program led to higher improvements on visuo-spatial working memory, visuo-spatial learning, and focused attention (see Table 3; Peretz et al., 2011). However, a comparison between commercial entertainment-focused games with serious brain-training games is hindered by the fact that the computer and video games were not originally designed as cognitive training tools. Task and factor analyses of computer games revealed inconclusive results about which underlying cognitive functions they exercised (Ackerman et al., 2010; Whitlock et al., 2012; Baniqued et al., 2013). One study compared the effects of Nintendo Wii training to a general reading assignment on fluid and crystallized intelligence and processing speed. Although the 15 Wii tasks could be assigned descriptively to different cognitive functions such as perceptual speed, working memory or spatial navigation, a factor analysis revealed only one underlying cognitive factor. Consequently, performance on all tasks was aggregated to form an overall composite score of training performance. While both reading and the Wii training resulted in significant improvements on the trained tasks, there were no transfer effects from either training condition to a cognitive test battery assessing fluid and crystallized intelligence and processing speed (see Table 3; Ackerman et al., 2010). Similarly, Whitlock et al. (2012) used a task analysis to identify the cognitive functions challenged by the video game World of Warcraft. This task analysis was based on the verbal protocol of two young novice and two young expert players. It revealed that the game challenged task switching and attentional control. In comparison to a passive control group, the training group improved performance on a measure of attentional control. This training-related transfer to an attentional control measure thus supported the result of the task analysis (see Table 3).

In sum, while extensive research on video and computer game training suggests that they may have beneficial effects on older adults' cognition with small to medium effect sizes, the range of transfer varies greatly (see Table 3). The big advantage of video game training is the complex nature of the training tasks, while the virtual environment nevertheless allows some experimental control over participants' reactions and performance.

### Summary: pros and cons of the three multi-domain training approaches

Multi-domain training studies have shown promising results regarding training-related improvements and transfer to various cognitive functions. Most studies were conducted with video game training and there is meta-analytic evidence for video game training to improve healthy older adults' memory, attention, and reaction time (Toril et al., 2014). Multi-domain training studies that introduced healthy older adults to novel leisure activities or a series of novel tasks revealed promising results, but these studies have been conducted less frequently and are more heterogeneous regarding the training tasks and their impact on cognition, impeding the ability to draw broad and systematic conclusions about which training conditions are most beneficial.

In contrast to paper-pencil training tasks (Cheng et al., 2012) or complex leisure activities (e.g., Experience Corps, Senior Odyssey, Acting, Synapse Project), computerized training has the advantage of providing individual feedback and adapting training task difficulty to individual performance levels, thereby maintaining a motivating and challenging learning experience during the entire training period (Green and Bavelier, 2008). The common open question of all the reviewed studies is which training component or combination of training components is responsible for the observed transfer. Due to the complex nature of the training regimes, oftentimes this cannot be directly inferred. There have been attempts to investigate the underlying cognitive functions addressed by computer and video game training (Ackerman et al., 2010; Whitlock et al., 2012; Baniqued et al., 2013), however, results have been inconclusive. Studies introducing novel leisure activities found certain activities to be more beneficial than others (Noice et al., 2004; Noice and Noice, 2009; Park et al., 2014). For example, Park et al. (2014) found acquisition of digital photography skills to be most effective with regard to transfer on episodic memory and visuo-spatial processing. However, digital photography courses took place in a group session. While social activities alone did not improve cognition, it remains open as to whether digital photography alone or its combination with socializing was the determining factor for transfer. Furthermore, we do not know which cognitive functions were challenged by digital photography. There is better control over the trained domains when training several tasks in series. Still, the unique contribution of each training domain cannot be determined in the available sequential training studies. Training-related improvements can be a result of the improvements on all functions equally, a greater improvement of one of the functions relative to others, or a result of the fact that the cognitive functions were trained one after the other (see discussion in Winocur et al., 2007a).

The simultaneous training of several cognitive functions not only trains each component function, but also the orchestration of these multiple functions. Furthermore, training regimes are supposed to be more effective when incorporating variable training conditions that challenge flexible information processing rather than supporting the development of specific strategies (Lustig et al., 2009; Karbach, 2014). There was one study that directly investigated whether the simultaneous training of several cognitive functions was different from the training of each of the functions in series (Neuroracer; Anguera et al., 2013). While the training of each function separately led to increases in both training tasks, the simultaneous training increased performance on each component task, the simultaneous training task, and additionally transferred to working memory and sustained attention. This finding is intriguing because dual-task training was not only more effective, but also more efficient since the overall training duration was the same for the simultaneous and the sequential training conditions.

It remains a matter of investigation to determine which cognitive functions are trained by multi-domain training and which of its components are necessary to enable transfer. Furthermore, there is a need for the development of more comprehensive theories about how transfer is achieved (cf. Noack et al., 2014). While the selection of transfer test batteries needs careful consideration, theory-driven development of training regimes is also crucial (cf. Noack et al., 2014). Serious games as custom-designed training tools offer one promising avenue, however, their development and application is still in the early stages (Anguera et al., 2013; Fua et al., 2013; Robert et al., 2014). They not only enable researchers to incorporate effective training elements, but also to embed training in a game-like environment that enhances motivation. We will next present the serious game Hotel Plastisse, which was designed to compare the simultaneous multi-domain training of three different cognitive functions with the training of each component function to better understand the processes underlying observed training and transfer effects, while at the same time providing an attractive and motivating training environment.

## The iPad-based training frame hotel plastisse

**Figure 1:**
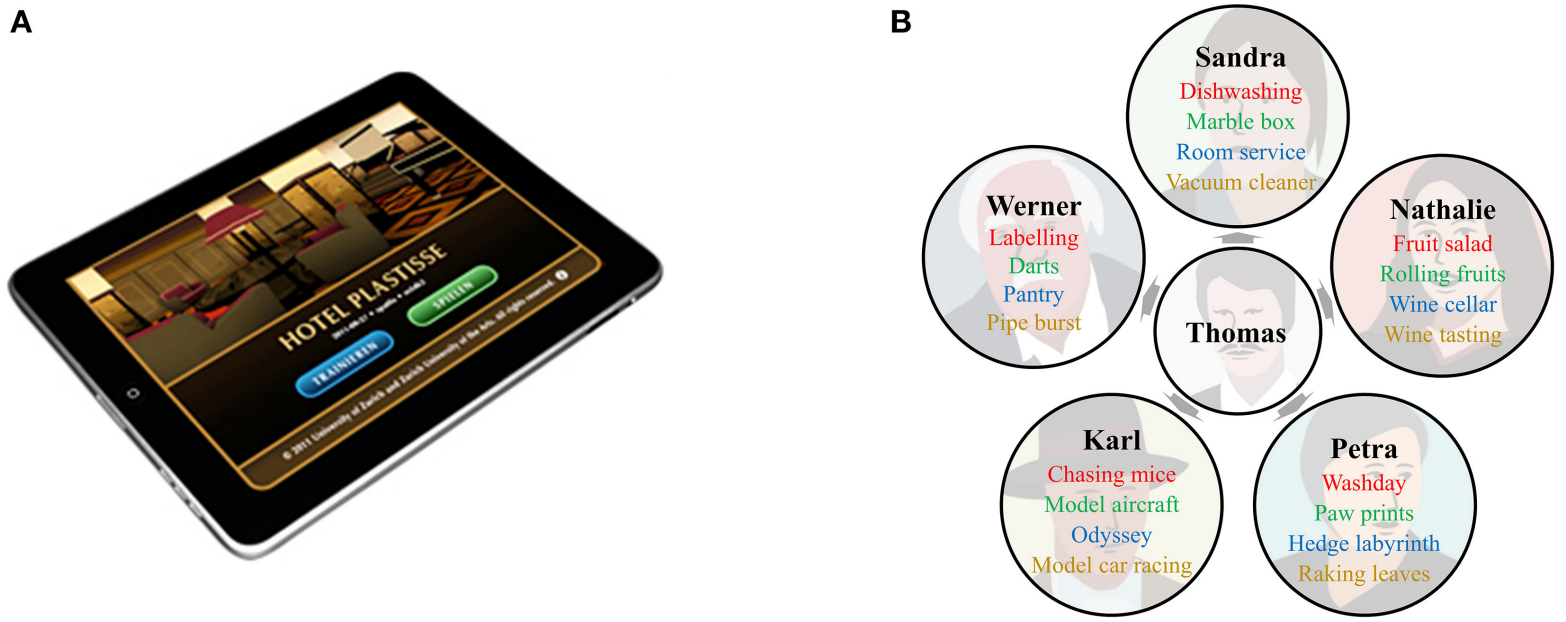
**Hotel Plastisse as an iPad-based serious training game**. **(A)** Start screen of the Hotel Plastisse app. **(B)** The training setting takes place in a hotel. The participant interacts with several avatars who are the same across training conditions (red = inhibition training tasks, green = visuomotor function training tasks, blue = spatial navigation training tasks, orange = multi-domain training tasks).

The aim of the iPad-based serious game Hotel Plastisse is a controlled comparison of multi-domain and single-domain cognitive training (see Figure 1A). Therefore, it allows the comparison of simultaneously training multiple cognitive functions to the training of each single cognitive function. According to principles suggested for effective training programs (Lövdén et al., 2010; Schmiedek et al., 2010a), the training is designed to be intense: it uses an adaptive algorithm to challenge individual performance levels optimally, consists of several different training tasks targeting the same function in order to minimize perception-based, task-specific strategies, provides individual performance-based feedback, and implements game elements to keep up motivation. Furthermore, an iPad-based training app has several advantages which are especially beneficial for training older adults: it does not require complicated technical knowledge, the touchscreen is clearly structured, easy to handle, and has a high resolution to maximize contrasts. In addition, the iPad allows unimanual and bimanual motor control in three dimensions, which extends typical computer-based applications. The small device allows participants to carry it along easily and thus flexibly integrate training into everyday life. At the same time, training is controlled by registering all training-related activities. Continuous transfer of the training data to a server enables training supervision and needs-oriented communication with the participants.

### Training conditions

The three single-domain conditions train inhibition, visuomotor function, or spatial navigation exclusively, while the multi-domain training trains these three cognitive functions *simultaneously*. These functions were selected such that they can be clearly separated in terms of task components. Furthermore, they refer to distinct cognitive processes that are affected by age-related cognitive decline and are associated with distinct neural networks (inhibition: Chambers et al., 2009; spatial navigation and spatial memory: Klencklen et al., 2012; visuomotor function: Lohse et al., 2014). Each training condition consists of five different training tasks, or minigames.

A training session includes the completion of all five minigames in a fixed, quasi-randomized order. Each minigame takes 6–10 min to complete, which results in a total session time of 45–60 min including instructions and feedback. All training conditions encompass 50 daily training sessions with adaptive task difficulty. The training parameters and settings between the multi-domain training and the single-domain training conditions are comparable. The difficulty level of the current training session depends on the performance of the previous training session: A score of 80% or higher results in a level increase for the subsequent training session, a score below 60% results in a level decrease, and a score between 60 and 80 percent results in maintenance of the current level. Training score protocols are transferred to a data server immediately after training completion to enable supervision of training progress by the researchers.

### Training setting

The training takes place in the virtual setting of a hotel. Several avatars interact with the participants, explain the training tasks, and give feedback. On the first day of the Hotel Plastisse training, the participant is greeted with a short written text explaining the background story. The main figure is Thomas who has recently opened a hotel. He is introduced as the participant's nephew. Since his barkeeper Daniel is sick, he needs assistance with the daily business of the hotel. Therefore, he asks the participant to help out.

At the beginning of each training session and in-between the different training tasks, participants interact with Thomas at the bar in the hotel lobby. He sends them to the hotel guest Karl and the employees Sandra (the maid), Nathalie (the cook), Werner (the superintendent), and Petra (the gardener). Each avatar is responsible for one training task in each training condition. The five avatars are the same across the different single-domain and multi-domain training conditions (see Figure 1B). They continuously lead the participants through the training by presenting written instructions and feedback in German. After each training task, participants walk through the hotel back to the lobby where they meet Thomas who sends them to the next employee. Over the course of the 50 training sessions, special events and feedbacks are interspersed unexpectedly to prevent boredom (e.g., virtual flowers as a thank-you gift for helping out with the hotel).

All events and scenes of a training session are accompanied by music and sound effects. The introductory scene with Thomas is accompanied by a piano piece. When walking through the hotel, participants hear the sound of footsteps and opening doors. The avatars interact with the participants through written texts that are typed in real-time accompanied by the sound of a typewriter. During the training task, there is a background sound and each visual feedback is supported by auditory feedback. This makes the training game more realistic, and also supports the awareness of the game events and feedback (e.g., different sounds for points and errors).

### Training procedure

Hotel Plastisse is started by pressing the “Hotel Plastisse” icon on the iPad. After an initial screen with the training name and the copyrights, the training participant is presented with options for “training” and “practice.” The practice tasks are only available during the first five training sessions. For both the training and the practice options, the participant has to log in with a personal code. This personal code is assigned by the study supervisor and defines which training condition is loaded. By logging in with a personal code, the participant's training profile is loaded and training continues based on the previously saved information from the last training session. Furthermore, each training protocol that is uploaded contains the personal code for later longitudinal training time-series mapping.

When participants choose the practice option, they are presented with a list of the five training tasks of the assigned training condition. When they choose a task to practice, a short extract is presented with the lowest difficulty level (Level 0). After each practice run, participants can choose another practice task, or start with the training.

**Figure 2:**
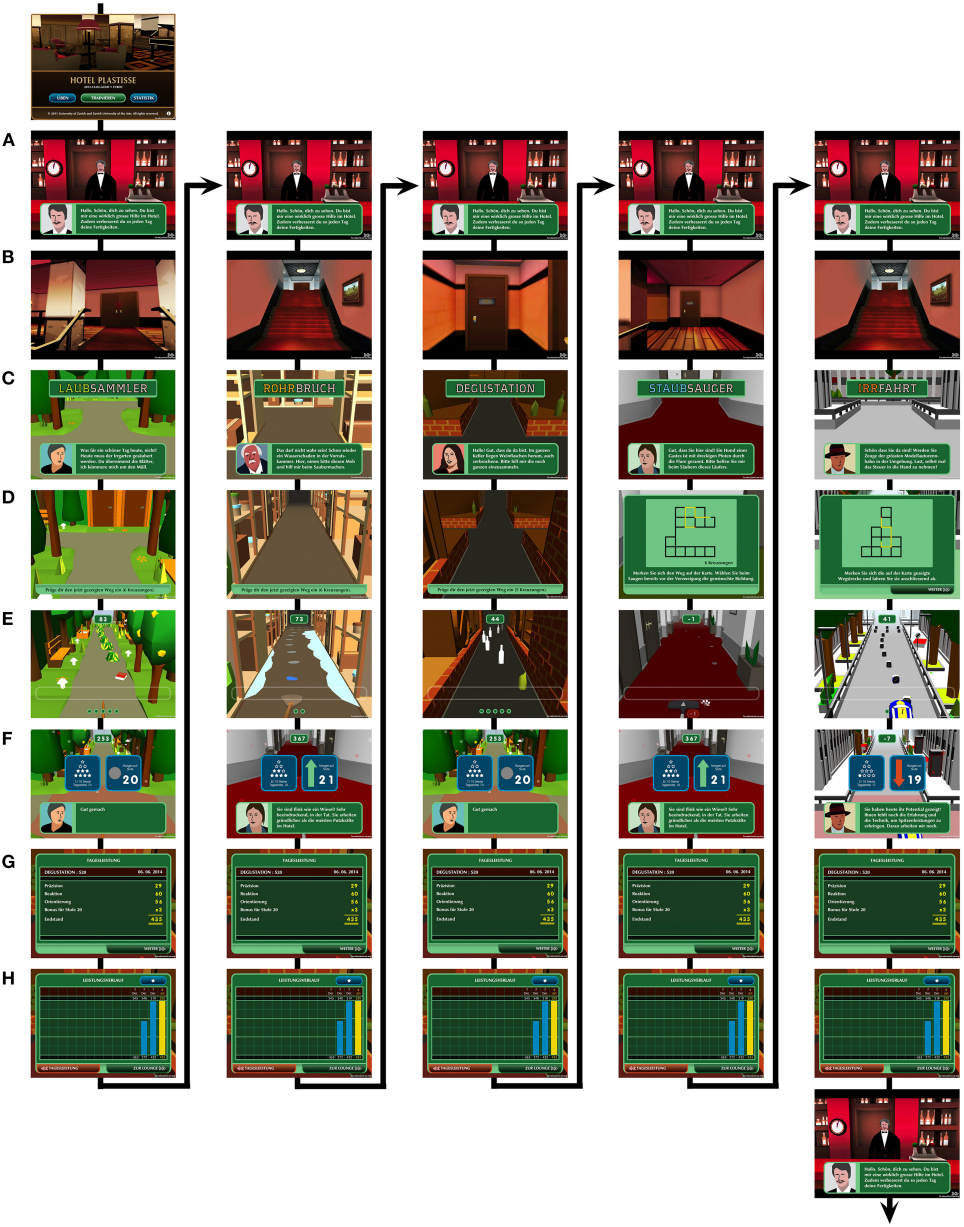
**Example of a multi-domain training session. (A)** At the beginning and in-between the five minigames, the participants interact with Thomas. **(B)** Participants walk through the hotel lobby and floors to one of the employees. **(C)** The respective employee provides the instructions for the training task. **(D)** The multi-domain training tasks requires memorizing a labyrinth by either a map (bird's eye condition) or an animated labyrinth (land mark condition). **(E)** The retrieval requires recalling the labyrinth by finding the correct path (always in the landmark condition). **(F)** At the end of each training task, percentage of performance and the level for the next training session are displayed. **(G)** This is followed by a detailed feedback. **(H)** At the end, the training course over the last 14 days is shown. This procedure **(A–H)** is repeated for all five training tasks.

When participants choose the training option, Thomas welcomes them to the training session at the hotel bar (see Figure 2A). The participant then walks from Thomas through the hotel lobby to the respective employee (e.g., to the kitchen, the garden, a guest's room; see Figure 2B). The employee greets and explains the training task (see Figure 2C). Written instructions are provided on two slides, the participant can press the forward buttons, there is no time-limit. When ready to start the training task, a countdown from three to one prepares the participant for the task.

During the training, immediate feedback is provided by visual and auditory feedback in form of a counter in which points are added or subtracted after each reaction. The counter is placed at the top of the screen to visualize the total score (see Figures 2D,E). Its function is to motivate and push participants toward their performance limits. After having completed a training task, the participant is presented the level for the next training session (see Figure 2F): An upward-pointing arrow indicates a level increase in the next training session, a downward-pointing arrow indicates a level decrease, and a circle indicates level maintenance for the next training session. Importantly, the percentage always reflects the ratio of correct to total responses (correct and incorrect) independent of the amount of points awarded for an individual game element. The percentage of performance is visualized with one to ten stars, each star reflecting 10% of the maximal score: 10 stars reflect a perfect performance of 100%, nine stars reflect a performance of 90–99%, and one star reflects a performance of 10–19%. The next feedback slide provides a detailed overview of the points (see Figure 2G) and the final slide shows the training course over the last 14 sessions (see Figure 2H). During the feedback period, a high score file is uploaded to the data server that contains a detailed protocol of the training session. After the feedback, participants are walked back through the hotel to the lobby. Thomas then sends the participants to the next employee in need of help. The same procedure is repeated for all five training tasks. After the last training task, Thomas bids the participant goodbye and the app closes automatically.

### Inhibition training

The inhibition training consists of five different go/no-go tasks with a task duration of 6 min. The game principle across the five training tasks is the same: A continuous stream of go and no-go stimuli is presented. Participants are supposed to tap on the touch screen for go stimuli and inhibit their reaction to no-go stimuli (the whole screen registers taps independent of the tapping location). Each correct response to a go stimulus results in a temporary buffer point. As soon as a no-go stimulus is ignored correctly, the temporary buffer points are transferred to the counter and an animated number of the number of transferred points appears, while the buffer points are lost when reacting to a no-go stimulus erroneously. In this case, an animated number shows the number of buffer points lost. Failure to react to a go stimulus results in no additional buffer point, but is not penalized otherwise (incorrect reaction to a go stimulus). At the end of each training task, overall feedback is provided in the form of the absolute number of points (end score of the counter, which is the total of transferred buffer points), the number of wrong reactions to no-go stimuli, and the percentage of the maximum score that could have been reached when performed on the task perfectly. The percentage of the maximum score determines the level for the subsequent training session (increase, decrease, or same level). This percentage is calculated as the number of correct responses divided by the sum of all correct and wrong responses. Therefore, the buffer does not influence the final score, but rather motivates participants to engage in the task. The difficulty of the levels is increased by decreasing the inter-stimulus delay.

#### Washday

**Figure 3:**
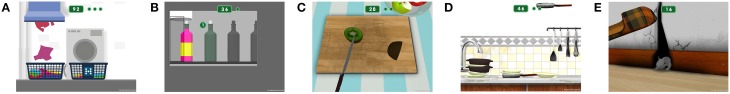
**Screenshots of the inhibition minigames**. **(A)** Washday, **(B)** Labeling, **(C)** Fruit salad, **(D)** Dishwashing, **(E)** Chasing mice.

In the washday minigame, participants help Petra sort laundry (see Figure 3A). The clothes are blown out of the drier on top of the screen and fall down toward two baskets. Clothes with a hotel logo have to be sorted to the basket with the logo (go stimuli), while clothes without a label are to be sorted into the other basket (no-go stimuli). Following a reaction to one piece of clothing, the baskets are moved such that it is sorted into the basket with the logo. Clothes without a logo are sorted to the correct basket when a reaction is suppressed correctly (no-go stimuli). There are several go and no-go stimuli per training session (different clothes such as pants, shirts, sweaters) with the logo as the identifying feature for go stimuli.

The delay between two stimuli is 1.72 s on Level 1 and is reduced by 0.03 or 0.02 s every time the difficulty level is increased, resulting in a delay of 0.40 s on Level 50. Since the task duration is fixed, the total amount of stimuli increases from 173 go stimuli and 36 no-go stimuli on Level 1 to 747 go stimuli and 153 no-go stimuli on Level 50. Every 3 to 6 go stimuli, a no-go stimulus appears. The practice task contains 28 go and 6 no-go stimuli with a delay of 1.75 s (see Table 4).

**Table 4:** **Adaptive training parameters of the inhibition minigames**.

	**Washday**			**Labeling**			**Fruit salad**			**Dishwashing**			**Chasing mice**		
	**Delay**	**Go**	**No-go**	**Delay**	**Go**	**No-go**	**Delay**	**Go**	**No-go**	**Delay**	**Go**	**No-go**	**Delay**	**Go**	**No-go**
Level 1	1.72	173	36	1.72	157	52	1.97	144	38	1.87	156	37	1.97	141	42
Level 10	1.48	202	41	1.46	185	62	1.70	167	44	1.62	180	42	1.66	167	50
Level 20	1.21	247	51	1.17	231	77	1.40	203	54	1.34	218	51	1.32	210	63
Level 30	0.94	318	65	0.88	307	102	1.10	259	69	1.06	275	65	0.98	283	84
Level 40	0.67	446	91	0.59	458	153	0.80	356	95	0.78	374	88	0.64	433	129
Level 50	0.40	747	153	0.30	900	300	0.50	569	151	0.50	583	137	0.30	924	276

*Across levels, the delay between two consecutive stimuli is shortened (in sec), which results in an increasing number of go and no-go stimuli at a fixed task duration of 6 min*.

#### Labeling

In the labeling minigame, participants help Werner label bottles (see Figure 3B). Bottles are presented in a continuous stream, transported on a conveyer belt from the left to the right side of the screen. Bottles to be labeled (go stimuli) are different in color or shape from bottles not to be labeled (no-go stimuli). At the beginning, participants are shown the type of bottle they have to label (go stimulus). While there is one specific go and no-go stimulus per training session, the bottles vary over the training sessions in shape and color. Following a reaction to a bottle, the labeling machine on the left side of the screen swoops down and labels the bottle.

The delay between two bottles is 1.72 s on Level 1 and is reduced by 0.02 or 0.03 s every time the difficulty level is increased, resulting in a delay of 0.30 s on Level 50. Since the task duration is fixed, the total amount of stimuli increases from 157 go stimuli and 52 no-go stimuli on Level 1 to 900 go stimuli and 300 no-go stimuli on Level 50. Every 3 to 6 go stimuli a no-go stimulus appears. The practice task contains 26 go and 9 no-go stimuli with a delay of 1.75 s and a minimum of 2 go stimuli before a no-go stimulus appears (see Table 4).

#### Fruit salad

In the fruit salad minigame, participants help Nathalie prepare a fruit salad in the kitchen (see Figure 3C). Fruits appear one by one on a cutting board in the middle of the screen. At the beginning, participants are shown which fruit to cut. While there is one specific go (fruit to be cut) and no-go stimulus (fruits not to be cut) per training session, the fruits vary over the training sessions (e.g. apples, kiwis, lemons, grapefruits, oranges). Following a reaction, a knife cuts the fruits in two halves.

The delay between two stimuli is 1.97 s on Level 1 and is reduced by 0.03 s every time the difficulty level is increased, resulting in a delay of 0.50 s on Level 50. Since the task duration is fixed, the total amount of stimuli increases from 144 go stimuli and 38 no-go stimuli on Level 1 to 569 go stimuli and 151 no-go stimuli on Level 50. Every 3 to 6 go stimuli a no-go stimulus appears. The practice task contains 24 go and 6 no-go stimuli with a delay of 2 s (see Table 4).

#### Dishwashing

In the dishwashing minigame, participants help Sandra stack plates and pots in the kitchen (see Figure 3D). Plates and pots move from the top to the bottom of the screen while participants have to pile them up on three different piles. The plates and pots that are not horizontally aligned have to be turned (go stimuli) while others appear already horizontally aligned (no-go stimuli). The plates and pots are continuously presented, with a plate/pot to be stacked on the left pile followed by a plate/pot to be stacked on the middle pile, and finally a plate/pot to be stacked on the right pile. The color of the plates and pots varies across levels and there are several go and no-go stimuli per training session with stimulus orientation as the identifying feature (horizontal alignment: no-go stimulus). Following a correct reaction to a go stimulus, the plates and pots are turned and piled up, while they burst when failed to turn (no reaction to go stimulus).

The delay between two stimuli is 1.87 s on Level 1 and is reduced by 0.03 or 0.02 s every time the difficulty level is increased, resulting in a delay of 0.50 s on Level 50. Since the task duration is fixed, the total amount of stimuli increases from 156 go stimuli and 37 no-go stimuli on Level 1 to 583 go stimuli and 137 no-go stimuli on Level 50. Every 3 to 6 go stimuli a no-go stimulus appears. The practice task contains 26 go and 6 no-go stimuli with a delay of 1.90 s and a minimum of 2 go stimuli before a no-go stimulus appears (see Table 4).

#### Chasing mice

In the chasing mice minigame, participants scare away mice in Karl's hotel room (see Figure 3E). The animals come out of a hole in the wall and disappear after a short time. Mice (go stimuli) have to be scared away with a slipper, while pets of other hotel guests (no-go stimuli) have to be spared. While there is one specific go and no-go stimulus per training session, the animals vary over the training sessions. Following a reaction, the respective animal is scared off.

The delay between two animals is 1.97 s on Level 1 and is reduced by 0.03 or 0.04 s every time the difficulty level is increased, resulting in a delay of 0.30 s on Level 50. Since the task duration is fixed, the total amount of stimuli increases from 141 go stimuli and 42 no-go stimuli on Level 1 to 924 go stimuli and 276 no-go stimuli on Level 50. Every 3 to 6 go stimuli a no-go stimulus appears. The practice task contains 23 go and 7 no-go Stimuli with a delay of 2 s (see Table 4).

### Visuomotor function training

The visuomotor function training consists of five training tasks to practice eye-hand coordination with a duration of 6 min each. These tasks are designed to train unimanual or bimanual hand or finger movements by aiming at targets as precisely as possible. In the two tasks with unimanual control, participants use their index finger to aim at targets as precisely as possible along the x-axis. In the three bimanual tasks, participants move the iPad in the 3D-room along the x-, y-, and z-axes. The primary game mechanic across the five training tasks is the same: participants are presented with a continuous stream of targets. Two points are awarded for hitting a target perfectly, one point is awarded for hitting a target, and one point is subtracted from the total score if failed to hit a target (exception minigame marble box; see the description below). Upon each hit or miss, immediate feedback is provided acoustically and visually by sound and animated numbers. The points of the animated numbers are continuously added to the counter. At the end of each training task, overall feedback is provided by the absolute number of points, the number of perfect hits, the number of hits, the number of misses, and the percentage of the maximum score that could have been reached if every target was hit. The percentage of hits (independent of their precision, i.e., independent of two- or one-point reactions) in relation to the total number of targets determines the level for the next training session (increase, decrease, or maintenance). Depending on the minigame, difficulty increases across levels by the parameters speed (delay as specified by the time frame between the presentation of two targets) or the size of the targets.

#### Paw prints

**Figure 4:**
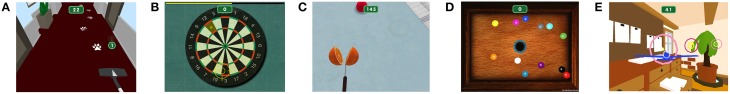
**Screenshots of the visuomotor minigames. (A)** Paw prints, **(B)** Darts, **(C)** Rolling fruits, **(D)** Marble box, **(E)** Model aircraft.

In the paw prints minigame, participants help Petra vacuum the hotel floor after a dog leaves dirty paw prints on the carpet (see Figure 4A). The vacuum cleaner is animated and vacuums at a level-specific speed. The participants have to aim at the paw prints as precisely as possible by moving the vacuum cleaner with their index finger on the screen (unimanual control, movements along the x-axis from left to right). Difficulty increases by the speed of the vacuum cleaner, which results in a reduced delay between two paw prints.

The delay between two paw prints is 1.18 s on Level 1 and is reduced by 0.01 or 0.02 s every time the difficulty level is increased, resulting in a delay of 0.35 s on Level 50. The practice task consists of a 1-min extract of the task with a delay of 1.20 s between two paw prints (see Table 5).

**Table 5:** **Adaptive training parameters of the visuomotor function minigames**.

	**Paw prints**	**Darts**	**Rolling fruits**	**Marble box**		**Model aircraft**
	**Delay**	**Scale**	**Delay**	**Speed**	**Marbles**	**Scale**
Level 1	1.18	1.05	1.48	1.01	2	0.98
Level 10	1.03	1.50	1.27	1.12	4	0.83
Level 20	0.86	2.00	1.04	1.24	6	0.66
Level 30	0.69	2.50	0.81	1.36	8	0.50
Level 40	0.52	3.00	0.58	1.48	10	0.33
Level 50	0.35	3.50	0.35	1.60	12	0.16

*Across levels, increasing (e.g., speed) or decreasing (e.g., scale) parameters require increased precision for unimanual and bimanual hand and finger movements. Delay is the reaction time frame between two targets (in sec). Scale = 1 represents the original size of 100% and is linearly up-scaled (Darts) or down-scaled (Model aircraft). Speed = 1 represents the original speed and is linearly up-scaled across levels (game-intern metric)*.

#### Darts

In the darts minigame, participants play darts with Werner (see Figure 4B). The aim is to throw the arrow onto the marked area on the dartboard. Participants control a crosshair by tilting the iPad (bimanual control). After the 4 s allotted to place the crosshair on the marked area, the arrow is thrown automatically to wherever the crosshair points. Difficulty increases by scaling down the size of the dartboard.

The game-internal scale represents the distance to the camera and is set to 1.05 on Level 1 and increases by 0.05 every time the difficulty level is increased, resulting in a scale of 3.50 on Level 50. Increasing the scale leads to a gradually smaller dartboard. The practice task consists of a 1-min extract of the task with a scale of 1 (see Table 5).

#### Rolling fruits

In the rolling fruits minigame, participants help Nathalie prepare a fruit salad (see Figure 4C). Different fruits (apples, kiwis, nectarines, oranges, and grapefruits) roll over a table from the top of the screen to the bottom. A knife on the bottom of the screen can be moved horizontally with the index finger (unimanual control). Fruits have to be cut in the middle. Difficulty increases by reducing the delay between two fruits.

The delay between two rolling fruits is 1.48 s on Level 1 and is reduced by 0.02 or 0.03 s every time the difficulty level is increased, resulting in a delay of 0.35 s on Level 50. The practice task consists of a 1-min extract of the task with a delay of 1.50 s between two rolling fruits (see Table 5).

#### Marble box

In the marble box minigame, participants play marbles with Sandra (see Figure 4D). The aim is to sink a target marble in the hole in the middle of the screen. The marbles are colored differently and the color of the target marble is indicated by a colored ring around the hole. Participants can move the marbles by tilting the iPad (bimanual control, movements are possible in all directions). Whenever the correctly colored marble is sunk, the next target marble has to be sunk (either the same or different color). The number of marbles remains the same during a minigame and sunk marbles are replaced. Sinking the correctly colored marble is awarded with one point, while sinking another marble is punished by subtracting one point from the total score. There is only the option to sink the correct or the wrong marble with no scale for precision. Difficulty increases by increasing the number and the moving speed of the marbles.

There are two marbles at Level 1. Every three to five difficulty levels, a marble is added, which results in a total of 12 marbles at Level 50. Level 1 starts with a speed of 1.01 (game-intern scale, increase of speed in percent), which is gradually increased by 0.01 or 0.02, resulting in a speed of 1.60 on Level 50. The practice task is a 1-min extract of the task with 2 marbles and a speed of 1 (ground speed; see Table 5).

#### Model aircraft

In the model aircraft minigame, participants steer Karl's model aircraft (see Figure 4E). The model aircraft flies at a fixed speed and is steered by tilting the iPad (bimanual control). The model aircraft has to be steered through rings, which are placed around the room in a circle of 20 rings, and every 3 s a new ring appears. There is an outer and an inner ring. Steering the model aircraft through the inner ring is awarded with two points, steering it through the outer ring is awarded with one point, and failing to fly through either ring is punished by subtracting a point from the total score. Difficulty increases by decreasing the size of the rings (scale parameter, game-internal scale with 1 as starting point), which requires more precise steering.

The scale of the rings is 0.98 on Level 1 and is reduced by 0.01 or 0.02 every time the difficulty level is increased, resulting in a scale of 0.16 on Level 50. The practice task is a 1-min extract of the task with a scale of 1 (see Table 5).

### Spatial navigation training

The spatial navigation training requires that participants memorize paths in labyrinths across five different training tasks. All tasks consist of an encoding and a retrieval phase. During encoding, 2D-maps (bird's eye perspective) or 3D-videos of labyrinths (landmark perspective) are presented. Retrieval always requires finding the memorized path in a 3D-labyrinth. During retrieval, participants have to decide on the correct direction at every crossroads. The decisions at the crossroads are either time-unlimited by choosing an arrow (unimanual control) or time-limited by tilting the iPad to the left, to the right, or not tilting it to move straight on (bimanual control; for a summary of the conditions for each minigame see Table 6).

**Table 6:** **Spatial navigation conditions for encoding and retrieval**.

		**Hedge labyrinth**	**Pantry**	**Wine cellar**	**Room service**	**Odyssey**
Encoding	Condition	Landmark	Landmark	Landmark	Bird's eye	Bird's eye
	Time	Limited	Limited	Limited	Unlimited	Unlimited
Retrieval	Condition	Landmark	Landmark	Landmark	Landmark	Landmark
	iPad Operation	Unimanual	Bimanual	Unimanual	Bimanual	Bimanual
	Decision time	Unlimited	Limited	Unlimited	Limited	Limited

*During encoding, participants either memorize a path in a labyrinth in the landmark or bird's eye perspective, while retrieval always takes place in the landmark perspective*.

The total training time per minigame is not exactly fixed to 6 min as it is in the two other single-domain training conditions for visuomotor function and inhibition due to the variability in time of encoding and retrieval and the increasing amount of time required for longer paths on higher levels. There are several different labyrinths available per training session.

A correct decision at a crossroads with three alternatives is awarded with two points, while a correct decision at a crossroads with two alternatives is awarded with one point. Wrong decisions are scored with zero points. Animated numbers show the points that are added to the counter. Following a wrong decision, the correct direction is indicated and the animation of the labyrinths continues in the correct direction. At the end of each training task, overall feedback is provided as the absolute number of points (end score of the counter), the number of correct decisions at the crossroads with two and with three alternative directions, the number of wrong decisions, and the percentage of correct decisions in relation to the total number of decisions. The percentage of correct decisions relative to all decisions determines the level of the next training session (increase, decrease, or same level). Across levels, difficulty increases by the length of the labyrinths.

Level 1 starts with a path consisting of 3 crossroads. Every six difficulty levels, a crossroads is added. From level 36, a crossroads is added every fourth level, which results in twelve crossroads for the levels 48–50 (see columns 4 of Table 7). Among the levels with the same number of crossroads, difficulty increases by the complexity of the labyrinths (i.e., the number of crossroads with three alternatives). The labyrinths are predefined for each level and randomly drawn from the respective pools (presentation of the same labyrinths across the minigames is minimized by counting the number of presentations). The practice task consists of two labyrinths with three crossroads.

**Table 7:** **Adaptive training parameters of the multi-domain minigames**.

	**Multi-domain minigame**																			
	**Raking leaves**				**Pipe burst**				**Wine tasting**				**Vacuum cleaner**				**Model car racing**			
**Inhi**	**Fruit salad**				**Chasing mice**				**Labeling**				**Washday**				Not comparable^*^			
**Visuo**	**Unimanual**				**Bimanual**				**Unimanual**				**Bimanual**				**Bimanual**			
**Spat**	**Hedge labyrinth**				**Pantry**				**Wine cellar**				**Room service**				**Odyssey**			
	**1**	**2**	**3**	**4**	**1**	**2**	**3**	**4**	**1**	**2**	**3**	**4**	**1**	**2**	**3**	**4**	**1**	**2**	**3**	**4**
Level 1	1.97	144	38	3	1.97	141	42	3	1.72	157	52	3	1.72	173	36	3	1.48	197	46	3
Level 10	1.70	167	44	4	1.66	167	50	4	1.46	185	62	4	1.48	202	41	4	1.30	224	53	4
Level 20	1.40	203	54	6	1.32	210	63	6	1.17	231	77	6	1.21	247	51	6	1.10	265	62	6
Level 30	1.10	259	69	8	0.98	283	84	8	0.88	307	102	8	0.94	318	65	8	0.90	324	76	8
Level 40	0.80	356	95	10	0.64	433	129	10	0.59	458	153	10	0.67	446	91	10	0.70	417	98	10
Level 50	0.50	569	151	12	0.30	924	276	12	0.30	900	300	12	0.40	747	153	12	0.50	583	137	12

*Upper part of the table: correspondence of each multi-domain minigame to the single domain minigames/conditions (inhi: inhibition; visuo: visuomotor function; spat: spatial navigation). The model car racing minigame has no corresponding inhibition minigame. Lower part of the table shows the parameters. These are comparable to the corresponding parameters of a single-domain training minigame as reported in Tables 4–6*.

* *Exception: The inhibition parameters of the model car racing minigame are not comparable to an inhibition minigame*.

*See description of the model car racing minigame. Column 1 shows the delay (in sec) between two stimuli (go/no-go stimuli and visuomotor targets, respectively), Column 2 shows the number of go, and column 3 the number of no-go stimuli per minigame. Column 4 shows the number of crossroads of a labyrinth at a particular level*.

#### Hedge labyrinth

**Figure 5:**
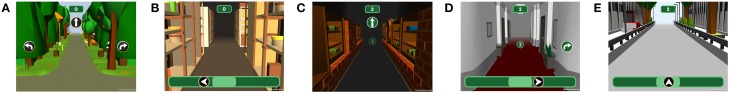
**Screenshots of the spatial navigation minigames. (A)** Hedge labyrinth, **(B)** Pantry, **(C)** Wine cellar, **(D)** Room service, **(E)** Odyssey.

In the hedge labyrinth minigame, participants help Petra find lost items (e.g., a purse; see Figure 5A). During the encoding phase, participants are walked through the hedge labyrinth (landmark perspective, time-limited encoding). During the retrieval phase, participants are walked through the same hedge labyrinth again. The animation is stopped at every crossroads and arrows pointing to the different directions are shown. The participants indicate the recalled direction by pressing the respective arrow (unimanual control). There is no time limit for choosing the direction. The animation time between two crossroads is 4 s.

#### Pantry

In the pantry minigame, participants help Werner find goods in the pantry (see Figure 5B). During the encoding phase, participants are walked through the pantry (landmark perspective, time-limited encoding). During the retrieval phase, participants are walked through the pantry again. The animation is not stopped at the crossroads, the participants indicate the recalled direction shortly before reaching a crossroads by tilting the iPad to the left for a left-hand turn, to the right for a right-hand turn, and keep it in horizontal position to keep on going straight (bimanual control). The animation time between two crossroads is 4 s.

#### Wine cellar

In the wine cellar minigame, participants help Nathalie find wine bottles ordered by the hotel guests (see Figure 5C). During the encoding phase, participants are walked through the wine cellar (landmark perspective, time-limited encoding). During the retrieval phase, participants are walked through the wine cellar again. The animation is stopped at every crossroads and arrows pointing to the different directions are shown. The participants indicate the recalled direction by the respective arrow (unimanual control). There is no time limit for choosing the direction. The animation time between two crossroads is 6 s.

#### Room service

In the room service minigame, participants help Sandra serve different guests (see Figure 5D). During the encoding phase, participants are presented a map of a labyrinth with a marked path from a starting to an end point (bird's eye perspective). Participants do not have a time limit to memorize the path. During the retrieval phase, participants are walked through the labyrinth. The animation is not stopped at the crossroads; the participants indicate the recalled direction shortly before reaching a crossroads by tilting the iPad to the left for a left-hand turn, to the right for a right-hand turn, and keep it in horizontal position to continue straight on (bimanual control). The animation time between two crossroads is 6 s.

#### Odyssey

In the odyssey minigame, participants play with Karl's model car (see Figure 5E). During the encoding phase, participants are presented a map of a labyrinth with a marked path from a starting to an end point (bird's eye perspective). Participants do not have a time limit to memorize the path. During the retrieval phase, participants drive with their model car through the labyrinth again. The animation is not stopped at the crossroads, the participants indicate the recalled direction shortly before reaching a crossroads by tilting the iPad to the left for a left-hand turn, to the right for a right-hand turn, and keep it in horizontal position to move straight on (bimanual control). The animation time between two crossroads is 6 s.

### Multi-domain training

The multi-domain training requires participants to *simultaneously* handle a spatial navigation task, an inhibition task, and a visuomotor function task. Therefore, the five multi-domain training tasks consist of two parts, accommodating requirements for the spatial navigation task: an encoding and a retrieval phase. During the retrieval phase of the spatial navigation task, participants have to simultaneously perform a visuomotor and an inhibition task.

During encoding, a path in a labyrinth is either presented in landmark or bird's eye perspective (similar to the single-domain spatial navigation training). During retrieval, participants have to decide on the correct direction at every crossroads (spatial navigation component; unimanual or bimanual control). The decision is always time-limited and the animation is not stopped. Between two crossroads, participants are presented with a continuous stream of go and no-go stimuli. Participants have to react to go stimuli and ignore no-go stimuli (inhibition task). In addition, the go-stimuli serve as visuomotor targets: these targets have to be hit as precisely as possible (unimanual or bimanual control; it is always the same control mode as the spatial navigation component requires for retrieval). While the timing of the reactions is critical for the inhibition task, their precision is critical to the visuomotor task.

Following a decision at a crossroads, participants are given feedback immediately. For a correct recall, a green arrow is shown pointing in the chosen direction. For a wrong recall, a red arrow is shown pointing in the chosen direction. A correct decision at a crossroads with three alternatives is awarded with more points than a crossroads with two alternatives (spatial navigation component; the points are higher compared to the spatial navigation training and increase across the difficulty levels to weight all three components equally). The points appear with animated numbers in a circle and are added to the counter at the top of the screen displaying the total score. Wrong decisions are scored with zero points. Between two crossroads, participants are supposed to tap on the touch screen for go stimuli and inhibit their reaction to no-go stimuli (inhibition component). Each correct response to a go stimulus results in a temporary buffer point. As soon as a no-go stimulus is ignored correctly, the temporary buffer points are transferred to the counter and an animated number of the transferred points appears, while the buffer points are lost when there was a wrong reaction to a no-go stimulus. Failure to react to a go stimulus results in no additional buffer point, but is not penalized otherwise (incorrect reaction to a go stimulus). Furthermore, two points are awarded for hitting a go stimulus perfectly, one point is awarded for hitting the go-stimulus slightly and one point is subtracted from the counter if failed to hit a go-stimulus (go-stimuli are targets for the visuomotor function component).

At the end of each minigame, overall feedback is provided in the form of the absolute number of points (end score of the counter), the number of points for each of the three components separately (inhibition component, visuomotor component, spatial navigation component), and the overall percentage of correct and incorrect reactions is presented (sum of all correct reactions for all components divided by all reactions). For the calculation of the percentage, the scoring is irrelevant. The scores provide feedback about the accuracy of each reaction only. The percentage of correct reactions determines the difficulty level of the next training session (increase, decrease, or same level). Task difficulty increases by decreasing the delay between go and no-go stimuli (inhibition and visuomotor components), the number of crossroads, and the complexity of the labyrinths (spatial navigation component). There is some variability in encoding duration when encoding time is unlimited. Therefore, the minigames are terminated after 6 min even when participants are not at the end of a retrieval phase. Time between two crossroads is 12 s for all minigames.

#### Raking leaves

**Figure 6:**
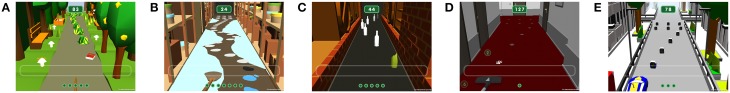
**Screenshots of the multi-domain minigames**. **(A)** Raking leaves, **(B)** Pipe burst, **(C)** Wine tasting, **(D)** Vacuum cleaner, **(E)** Model car racing.

In the raking leaves minigame, participants help Petra rake leaves in the hedge labyrinth (see Figure 6A). During the encoding phase, participants are walked through the hedge labyrinth (landmark perspective, time-limited encoding). During the retrieval phase, participants are walked through the same hedge labyrinth again. Before every crossroads, participants are shown leaves on the left, in the middle, and on the right side of the road. To indicate the direction, participants have to choose the corresponding leaf: the left leaf to turn left, the middle leaf to go straight, and the right leaf to turn right. Between the crossroads, participants have to pick up leaves (go stimuli), but ignore garbage (no-go stimuli). They have to react or inhibit their reaction as soon as the object is in a sensitive area indicated by a white rectangle. In addition, participants are supposed to aim at the leaves (visuomotor targets) as precisely as possible with their index finger (unimanual control).

The minigame raking leaves is structurally identical to the minigame hedge labyrinth of the spatial navigation training and difficulty increases in the same way by the number of crossroads and labyrinth complexity. The inhibition component uses the parameters of the inhibition minigame fruit salad (see Table 7 for a summary of the parameters across difficulty levels).

#### Pipe burst

In the pipe burst minigame, participants help Werner clean up water in the pantry caused by a pipe burst (see Figure 6B). During the encoding phase, participants are walked through the pantry (landmark perspective, time-limited encoding). During the retrieval phase, participants are walked through the same labyrinth again. Before every crossroads, participants are shown wet spots on the left side, in the middle, and on the right side. To indicate the direction, participants have to choose the corresponding wet spot: the left wet spot to turn left, the middle wet spot to go straightforward, and the right wet spot to turn right. Between the crossroads, participants have to clean up the wet spots (go stimuli), but ignore the oil slicks (no-go stimuli). They have to react or inhibit their reaction as soon as a wet spot or an oil slick is in the sensitive area displayed with a white rectangle. In addition, participants are supposed to aim at the wet spots (visuomotor targets) as precisely as possible by tilting the iPad (bimanual control).

The pipe burst minigame is structurally identical to the pantry minigame of the spatial navigation training and difficulty increases in the same way by the number of crossroads and labyrinth complexity. The inhibition component uses the parameters of the chasing mice inhibition minigame (see Table 7 for a summary of the parameters across difficulty levels).

#### Wine tasting

In the wine tasting minigame, participants help Nathalie put away wine bottles opened during a wine tasting (see Figure 6C). During the encoding phase, participants are walked through the wine cellar (landmark perspective, time-limited encoding). During the retrieval phase, participants are walked through the same wine cellar again. Before every crossroads, participants are shown a wine bottle on the left, in the middle, and on the right side. To indicate the direction, participants have to choose the corresponding wine bottle: the left wine bottle to turn left, the middle wine bottle to go straightforward, and the right wine bottle to turn right. Between the crossroads, participants have to collect the closed wine bottles (go stimuli), but to ignore the broken wine bottles (no-go stimuli). They have to react or inhibit their reaction as soon as the object is in the sensitive area displayed with a white rectangle. In addition, participants are supposed to aim at the closed wine bottles (visuomotor targets) as precisely as possible with their index finger (unimanual control).

The wine tasting minigame is structurally identical to the wine cellar minigame of the spatial navigation training and difficulty increases in the same way by the number of crossroads and labyrinth complexity. The inhibition component uses the parameters of the labeling inhibition minigame (see Table 7 for a summary of the parameters across difficulty levels).

#### Vacuum cleaner

In the vacuum cleaner minigame, participants help Sandra vacuum the hotel floor (see Figure 6D). During the encoding phase, participants are presented a map of the hotel floor showing a marked path from a starting to an end point (bird's eye perspective). Participants do not have a time limit to memorize the path. During the retrieval phase, participants are walked through the hotel again. Before every crossroads, participants are shown paw prints on the left side, in the middle, and on the right side. To indicate the direction, participants have to choose the corresponding paw print: the left paw print to turn left, the middle paw print to go straightforward, and the right paw print to turn right. Between the crossroads, participants have to vacuum the dry paw prints (go stimuli), but to ignore the wet paw prints (no-go stimuli). They have to react or inhibit their reaction as soon as a paw print is in the sensitive area displayed with a white rectangle. In addition, participants are supposed to aim at the dry paw prints (visuomotor targets) as precisely as possible by tilting the iPad (bimanual control).

The vacuum cleaner minigame is structurally identical to the room service minigame of the spatial navigation training and difficulty increases in the same way by the number of crossroads and labyrinth complexity. The inhibition component uses the parameters of the washday inhibition minigame (see Table 7 for a summary of the parameters across difficulty levels).

#### Model car racing

In the model car racing minigame, participants play with the model car of the hotel guest Karl (see Figure 6E). During the encoding phase, participants are presented a map showing a marked path from a starting to an end point (bird's eye perspective). Participants do not have a time limit to memorize the path. During the retrieval phase, participants drive with the model car through the same labyrinth again. Before every crossroads, participants are shown cans on the left side, in the middle, and on the right side. To indicate the direction, participants have to choose the corresponding can: the left can to turn left, the middle can to go straightforward, and the right can to turn right. Between the crossroads, participants have to hit the cans marked with a green tick (go stimuli), but ignore the cans marked with a red cross (no-go stimuli). They have to react or inhibit their reaction as soon as a can is in the sensitive area displayed with a white rectangle. In addition, participants are supposed to aim at the cans with a green tick (visuomotor targets) as precisely as possible by tilting the iPad (bimanual control).

The model car racing minigame is structurally identical to the odyssey minigame of the spatial navigation training and difficulty increases in the same way by the number of crossroads and labyrinth complexity, while the inhibition parameters are not comparable to an inhibition minigame (see Table 7 for a summary of the parameters across difficulty levels). The delay between two cans is 1.48 s on Level 1 and is reduced by 0.02 s every time the difficulty level is increased resulting in a delay of 0.50 s on Level 50. The total amount of stimuli increases from 197 go stimuli and 46 no-go stimuli on Level 1 to 583 go stimuli and 137 no-go stimuli on Level 50. Every 3 to 6 go stimuli a no-go stimulus appears. The practice task contains 32 go and 8 no-go stimuli with a delay of 1.5 s.

### Technical development and specifications

The training software was developed as an app for iPad versions 2 and 3. It was programmed with the commercial Unity 3D game engine, a platform for video game development (http://unity3d.com/). The International Normal Aging and Plasticity Imaging Center (INAPIC) of the University of Zurich (Zöllig et al., 2011) approached the Specialization in Game Design, Zurich University of the Arts (ZHdK; Prof. Ulrich Götz), with the project idea to develop single-domain and multi-domain training games for the three selected cognitive domains. To ensure clear and non-overlapping operationalization of each domain and of their combination, the developmental process was based on scientific requirements formulated by the INAPIC, while at the same time the ZHdK's Game Design group contributed their expertise in the area of serious game design. The ZHdK developed the overall serious game concept and design, and also performed the programming based on the scientific psychological requirements formulated by the INAPIC team. In the process of game development, the individual game components and minigames were further refined in close collaboration between INAPIC and ZHdK to match both cognitive psychological and serious game criteria. Initial designing and programming took place from beginning 2010 through mid-2012. Hotel Plastisse was tested in several steps by members of the INAPIC, ZHdK, and healthy older subjects of the target population who gave extensive feedback.

#### Configuration file

The training groups are defined with a fixed, quasi-randomized order of the training tasks for each of the 50 training sessions in the configuration file. The participants' personal code assigns them to one of the training groups.

#### Profiles

When the participants first log in with their personal code, a profile file is created named after the personal code consisting of five random letters. The profile file is saved on the participants' iPad and on the data server. It saves the training progress and consists of the training sessions, level, name of the high score files, and the final result of the current training session. This profile file is loaded when participants log in for the next training session and thereby enables to present them their individual level and training course. In addition to the participants' personal codes, there are general logins to present or test the training tasks at a specific level.

#### Data server

The data server contains the profile and high score files, which are uploaded after completion of each training task. For training supervision, a website shows the data files that are uploaded with the participants' code, the date, the training game, and the percentage of performance.

#### High score files

The high score files are text files containing the training protocol of a training task. For each event in the training task (e.g., reaction to a go stimulus, decision at a crossroads), the particular event, the correct reaction and the participant's reaction are recorded with a timestamp. In addition, the training session, the difficulty level, and the end score as percentage correct are saved in the high score files.

#### Bug fixing

Reported errors during the training can be fixed by the programmer. An updated version is downloaded automatically when the participants log in the next time. However, this is true only for errors that do not require a fundamental change of the software (e.g., a new version of the build). Software changes that require a new version can only be achieved by deinstalling the old and installing the new version.

## Discussion and outlook

The serious game Hotel Plastisse is an iPad-based training tool that aims at extending the understanding of multi-domain cognitive training. It allows the comparison of a multi-domain cognitive training to the training of each of its components. As an iPad-based training game, Hotel Plastisse has the advantage that participants can train flexibly in their home environment. There is no need to schedule training sessions in a laboratory, which allows high density of training and is feasible for participants who are restricted in mobility or live further away. Mobile data transfer enables some control over training by transferring training progress and the exact training time. However, participants are responsible for planning their training sessions and integrating them in their everyday life. One cannot control for their training environment or unexpected interruptions unless they report it in a diary. Social contact is usually important to older participants and the impact of its absence should be considered carefully for participants' motivation. However, there is always the possibility of organizing group events or scheduling regular contact with the experimenter over email and telephone, and to add a short daily training diary in either paper or electronic form.

The effectiveness of the Hotel Plastisse training needs to be addressed in a training study including pretest, posttest, and follow-up measurements with a transfer test battery in order to examine how single-domain training compares to multi-domain training in terms of pure training and, more importantly, transfer effects (see, Binder et al., under review). The advantage of Hotel Plastisse over other complex training tasks such as leisure activities or computer games not specifically designed for training is that each game event and response are registered and saved to the training protocol (high score file). This allows researchers to decompose overall training performance of the multi-domain training into performance on each of its components, analyze how performance on each component changes over training and how performance of each component relates to outcome measures. Depending on the research question, Hotel Plastisse can be integrated into different longitudinal study designs. Structural and functional neuroimaging would provide further insights into the mechanisms of multi-domain cognitive training (Lövdén et al., 2010). Additional control conditions such as iPad usage (Chan et al., 2014) or social activities (Park et al., 2014) could be interesting.

The Hotel Plastisse software can be adapted to a certain extent. Relative easy changes include changing training duration, number of minigames per training session, training algorithm for level increases and decreases, or the combination of minigames of different training conditions. It is not possible, however, to make structural changes to the multi-domain training such as adding an additional training domain (e.g., working memory) since the multi-domain training tasks administer the three training domains inhibition, visuomotor function, and inhibition simultaneously. Furthermore, there are some technical limitations due to the iPad platform. One limitation is the refresh rate of the iPad which constrains the accuracy of stimulus presentation and recording of reaction times. Another limitation is that available iPad memory restricts the presentation of presenting stimuli that are computationally intensive.

We believe that serious games provide a fascinating possibility to develop custom-designed training regimes for healthy older adults that are easily implemented in everyday life and at the same time approximate it to some degree. We hope to provide new insights into training healthy older adults' cognition with novel technologies and how age-related declines can be countered effectively in order to maintain cognitive functioning and overall quality of life. It remains a matter of empirical investigation to determine if multi-domain training is effective, which cognitive functions are targeted by multi-domain cognitive training, and how transfer to functions affected by older adults' cognitive decline and everyday life can best be achieved.

## Author contributions

JB conducted the literature search, documented the software, and wrote the manuscript with support of SS. JZ, AE, SM, CR, SS, LJ, and MM commented on the manuscript. The following members of the core INAPIC team were involved in developing the original research idea and initiating the collaboration with the Zurich University of the Arts (ZHdK): AE, LJ, MM, SM, CR, JZ. AE and JZ had the project lead of the software development. CR supervised the cooperation. LJ and MM were responsible for raising the funding. JB was in close exchange with the programmer, suggested and tested improvements based on first experiences.

### Conflict of interest statement

The authors declare that the research was conducted in the absence of any commercial or financial relationships that could be construed as a potential conflict of interest.
